# Geochemical and organic petrographic characteristics of high bituminous shales from Gurha mine in Rajasthan, NW India

**DOI:** 10.1038/s41598-020-78906-x

**Published:** 2020-12-17

**Authors:** Alok K. Singh, Mohammed Hail Hakimi, Alok Kumar, Adeeb Ahmed, Nor Syazwani Zainal Abidin, Mostafa Kinawy, Osama El Mahdy, Aref Lashin

**Affiliations:** 1 Petroleum Engineering and Geological Sciences Division, Rajiv Gandhi Institute of Petroleum Technology, Jais, Amethi 229 304 India; 2 Geology Department, Faculty of Applied Science, Taiz University, 6803 Taiz, Yemen; 3 Department of Earth and Environmental Sciences, Bahria University, Islamabad, 44000 Pakistan; 4 Geosciences Department, Faculty of Science and Information Technology, Universiti Teknologi PETRONAS, 32610 Bandar Seri Iskandar, Perak Malaysia; 5 Department of Geology, University of Malaya, 50603 Kuala Lumpur, Malaysia; 6 Petroleum and Natural Gas Engineering Department, College of Engineering, King Saud University, P.O. Box 800, Riyadh, 11421 Saudi Arabia

**Keywords:** Planetary science, Geochemistry, Petrology

## Abstract

A high bituminous shale horizon from the Gurha mine in the Bikaner sub-basin of the Rajasthan District, NW India, was studied using a collection of geochemical and petrological techniques. This study investigated the nature and environmental conditions of the organic matter and its relation to the unconventional oil-shale resources of the bituminous shale. The analyzed shales have high total organic carbon and total sulfur contents, suggesting that these shale sediments were deposited in a paralic environment under reducing conditions. The dominant presence of organic matter derived from phytoplankton algae suggests warm climatic marine environment, with little connection to freshwater enhancing the growth of algae and other microorganisms. The analyzed bituminous shales have high aquatic-derived alginite organic matters, with low Pr/Ph, Pr/*n*-C_17_, and Ph/*n*-C_18_ ratios. It is classified as Type II oil-prone kerogen, consistent with high hydrogen index value. Considering the maturity indicators of geochemical T_max_ (< 430 °C) and vitrinite reflectance values less than 0.40%VRo, the analyzed bituminous shale sediments are in an immature stage of the oil window. Therefore, the oil-prone kerogen Type II in the analyzed bituminous shales has not been cracked by thermal alteration to release oil; thus, unconventional heating is recommended for commercial oil generation.

## Introduction

**Figure 1:**
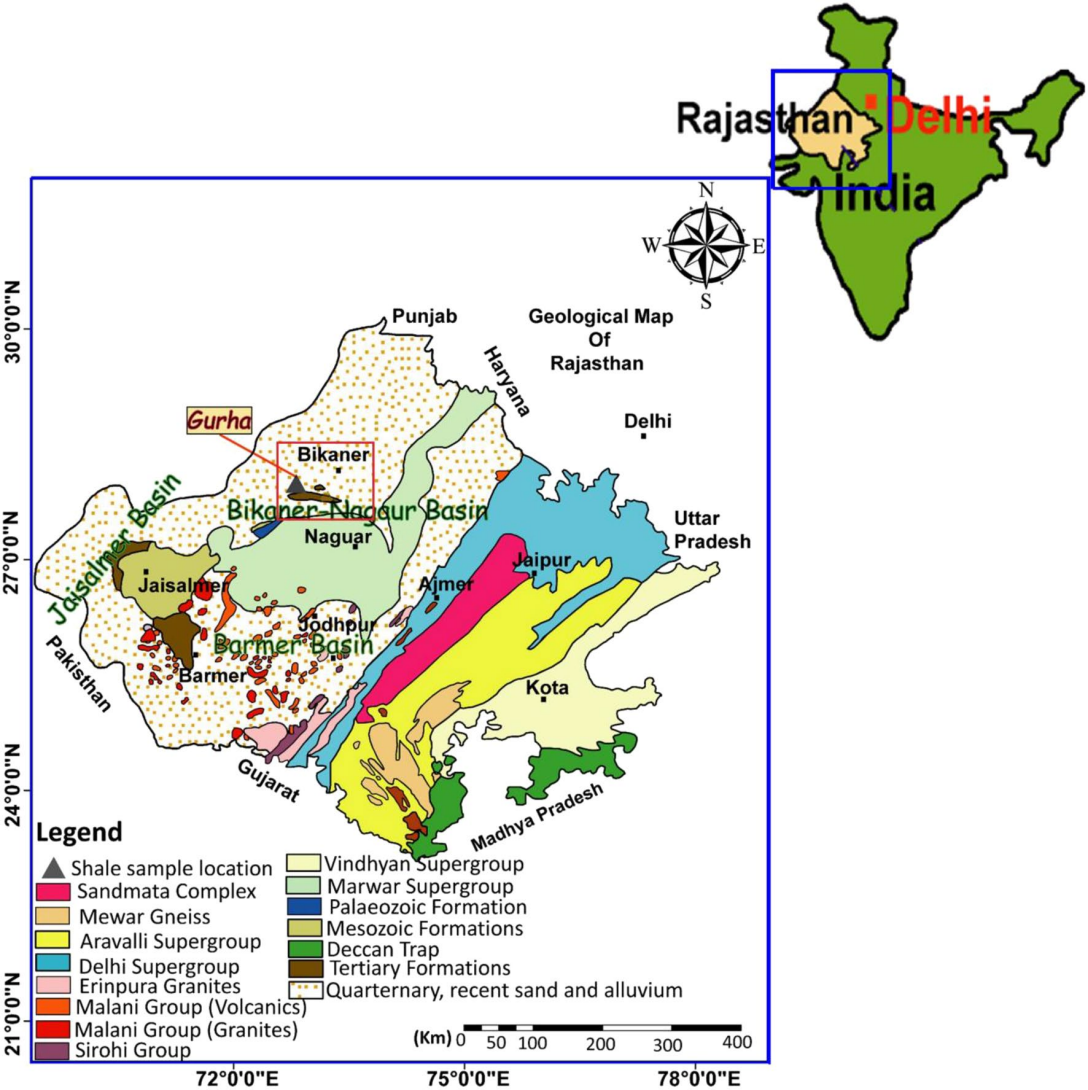
Geological map of Rajasthan showing the Gurha mine location in the Bikaner-Nagaur Basin, northwestern India. Arc Map GIS software 10.2.2 is used to create this figure, http://www.esri.com.

**Figure 2:**
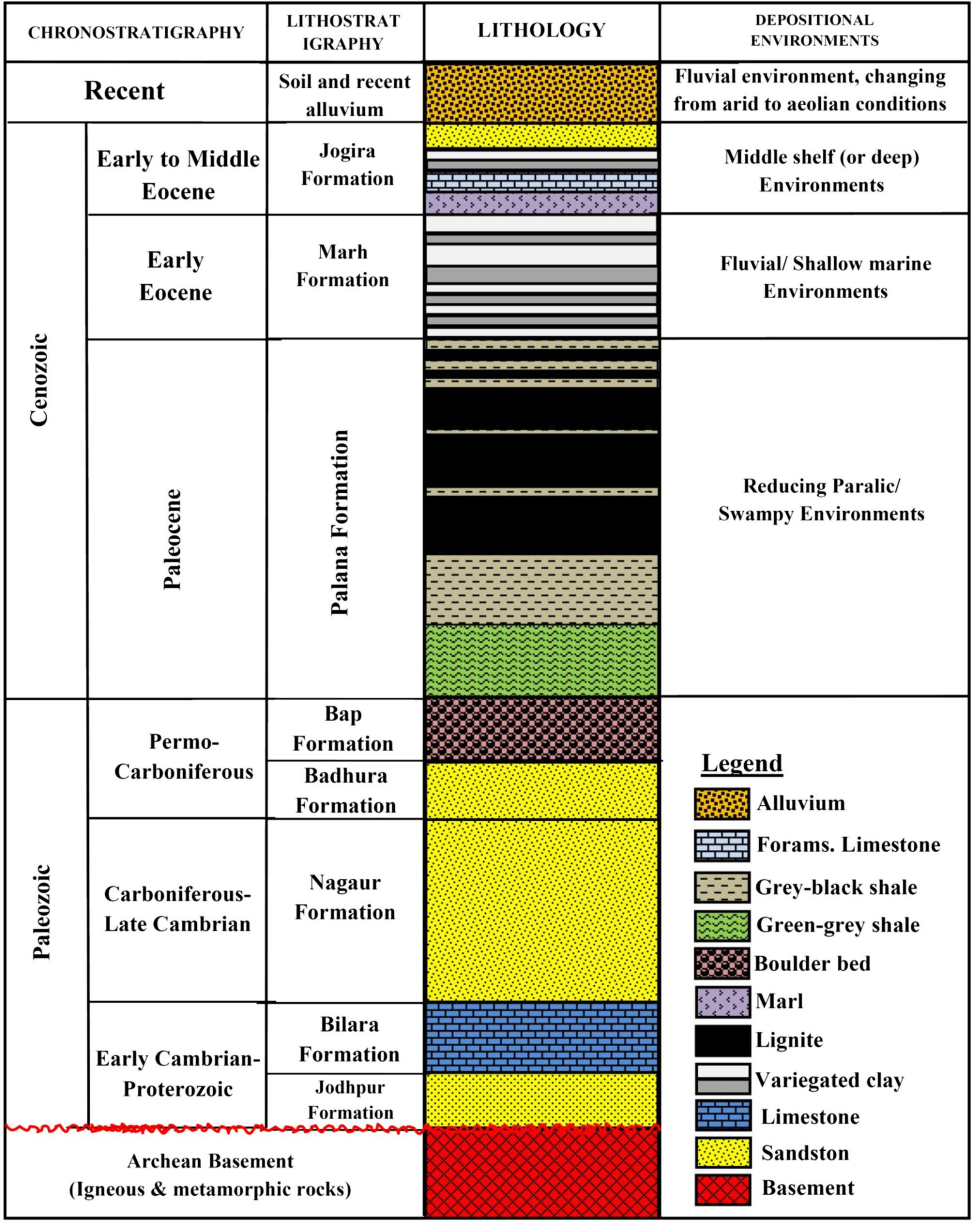
Generalized lithostratigraphic succession of the Bikaner-Nagaur Basin, northwestern India (modified after Mathews et al.^2^).

The Rajasthan state comprises three distinct basins, i.e., the Bikaner-Nagaur Basin, Jaisalmer Basin, and Barmer Basin (Fig. 1), related to the intracratonic sedimentation during the early Archean to Holocene period^1,2^. There are thick late Palaeozoic deposits in the Bikaner-Nagaur Basin followed by relatively thin Mesozoic and Cenozoic sediments (Fig. 2). The Cenozoic deposits are extended to more than 1700 km^2^. The Bikaner sub-basin of Rajasthan state constitutes the richest lignite deposits in northwestern India, mainly developed and explored at the Gurha and Barsingsar mines (Fig. 1).

The Gurha mine is of interest in this study and located between 27°52′ 35″ N latitude and 72°52′ 17″ E longitude, and situated in the Bikaner district of Rajasthan, India (Fig. 1). The Gurha mine comprises lignite and bituminous shale deposits, associated with the Palana Formation that accumulated during the Palaeocene age (Fig. 2)^3,4^. The lignite deposits and shale-bearing horizons in the Gurha mine have different thicknesses, with approximate 21 m of total sequence thickness. However, the lignite deposits of the Gurha mine received unprecedented attention from various academic researchers to understand the palaeo-vegetation and palaeo-environment^2–8^. The hydrocarbon generation potential from the lignite deposits in the Bikaner sub-basin of Rajasthan state was also investigated by^3,9,10^. In parallel with previous studies of the hydrocarbon source rock generation in the lignite deposits, this study focuses on the bituminous shale horizons in the Palana Formation, providing a bigger picture for the exploration of unconventional resources.

**Figure 3:**
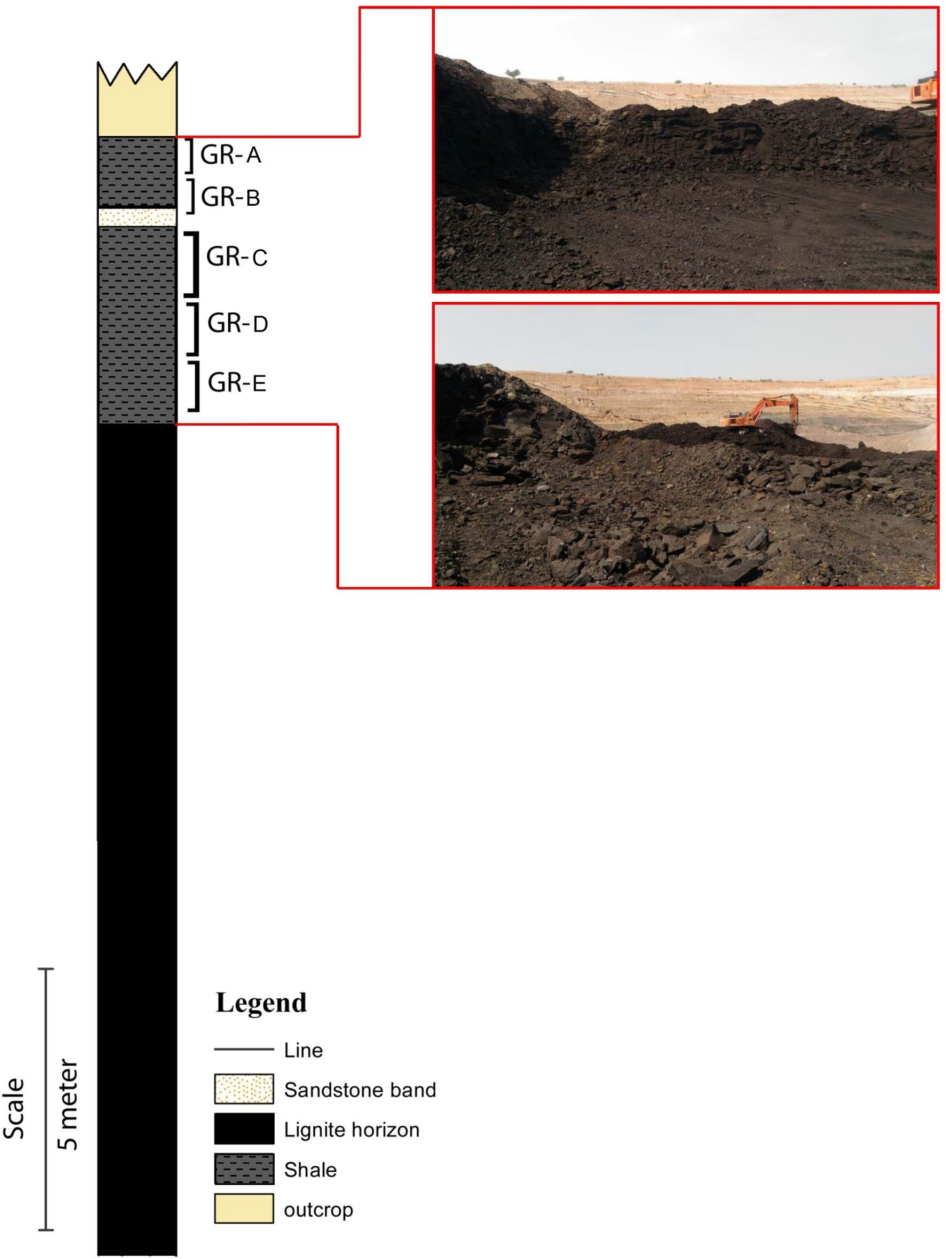
Lithology of the studied Gurha mine section, including the studied bituminous shale horizons.

This study aims to conduct the first detailed geochemical and petrographic characterization of the bituminous shale horizons of the Palana Formation exposed at the Bikaner sub-basin of Rajasthan state (Figs. 1 and 3). We aim to evaluate the richness of organic matter, the type of organic facies, and their connection to the oil-shale as an alternative unconventional energy resource using total organic carbon (TOC%) content, qualitative and quantitative pyrolysis analyses, and kerogen microscopic studies. Furthermore, to elucidate the origin and environmental conditions of the organic matter, the lipid biomarkers of the aliphatic fraction are integrated into the geochemical and petrological data.

## Geological setting

As a portion of the Indian shield, the Rajasthan state comprises sedimentary records spanning a period from Archean to Holocene^2^. Because of intracratonic sedimentation, three distinct basins, namely the Bikaner-Nagaur Basin, Jaisalmer Basin, and Barmer Basin (Fig. 1) have developed in the Rajasthan state^1^. The Bikaner-Nagaur Basin is a shallow intracratonic basin developed because of Malani magmatism^11^, has an elongated shape, and falls in the Nagaur and Bikaner districts of Rajasthan (Fig. 1).

Figure 2 presents the generalized stratigraphic succession of the Bikaner-Nagaur Basin. The Archean age succession functions as the basement rocks of the basin are followed by a thick Paleozoic and relatively thin Mesozoic and Cenozoic sedimentary sequences (Fig. 2), reaching a thickness of approximately 2100 m in the basin^12^. The Paleozoic rock sequence consists of the Jodhpur, Bilara, and Nagaur formations followed by the Badhura and Bap Formations, mainly composed of clastic and rocks (sandstone and shale) with additional sediments of carbonate (Fig. 2). The rock of the Bilara formation predominantly consists of limestone and dolomite with an algal structure. The Nagaur Formation comprises sandstone gypsum and clay pockets.

The Permo-Carboniferous age rocks of the Bap and Badhura formations consist of glacial drift deposits, including boulders, cobbles, and erratic rock deposits. The lignite-bearing Palana Formation of the early Palaeocene age, followed by the late Palaeocene-middle Eocene deposits of Marth and Jogira Formations (Fig. 2) initiated the Cenozoic era. The Palana Formation consists of lignite, bituminous shale, and grey shale horizons, deposited in different depositional environments ranging from reducing paralic settings to swampy environmental conditions^2,4^. The Marh Formation follows the Palana Formation and consists of argillaceous and ferruginous facies deposited in Fluvial to shallow marine environments (Fig. 2). The Marh sediments ended up with the deposition of Jogira Formation having a marine setting^2^. The investigation area is covered by recent deposits of aeolian sands (Fig. 2).

## Results

### Organic matter characteristics under the reflected light microscope

**Figure 4:**
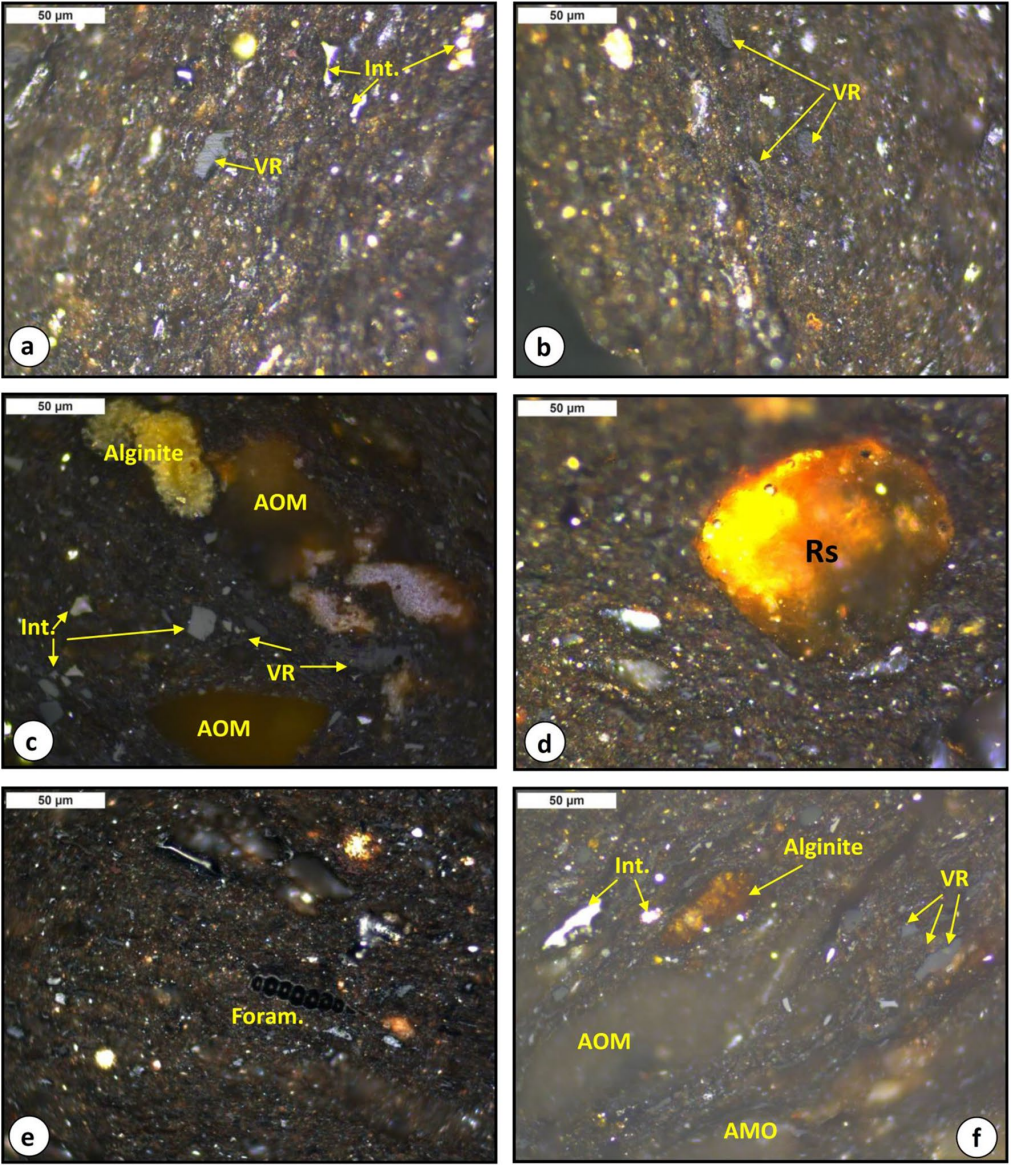
Photomicrographs of organic matter assemblage from the analyzed bituminous shales in the Paleocene Palana Formation; under reflected light, field width = 0.2 mm: (**a–d**) vitrinite (VR) and inertinite (Int.) organic matter associated with clay matrix, (**c**) mixed of organic matter, including alginite (*Botryococcus* ), amorphous organic matter (AOM), vitrinite (VR) and inertinite (Int.) organic matter, (**d**) other liptinitic organic matter (resinite (Rs)), (**e**) planktonic foraminifera associated with organic matter and clay matrix, and (**f**) mixed of organic matter, including alginite (*Botryococcus*), AOM, vitrinite (VR) and inertinite (Int.) organic matter.

**Figure 5:**
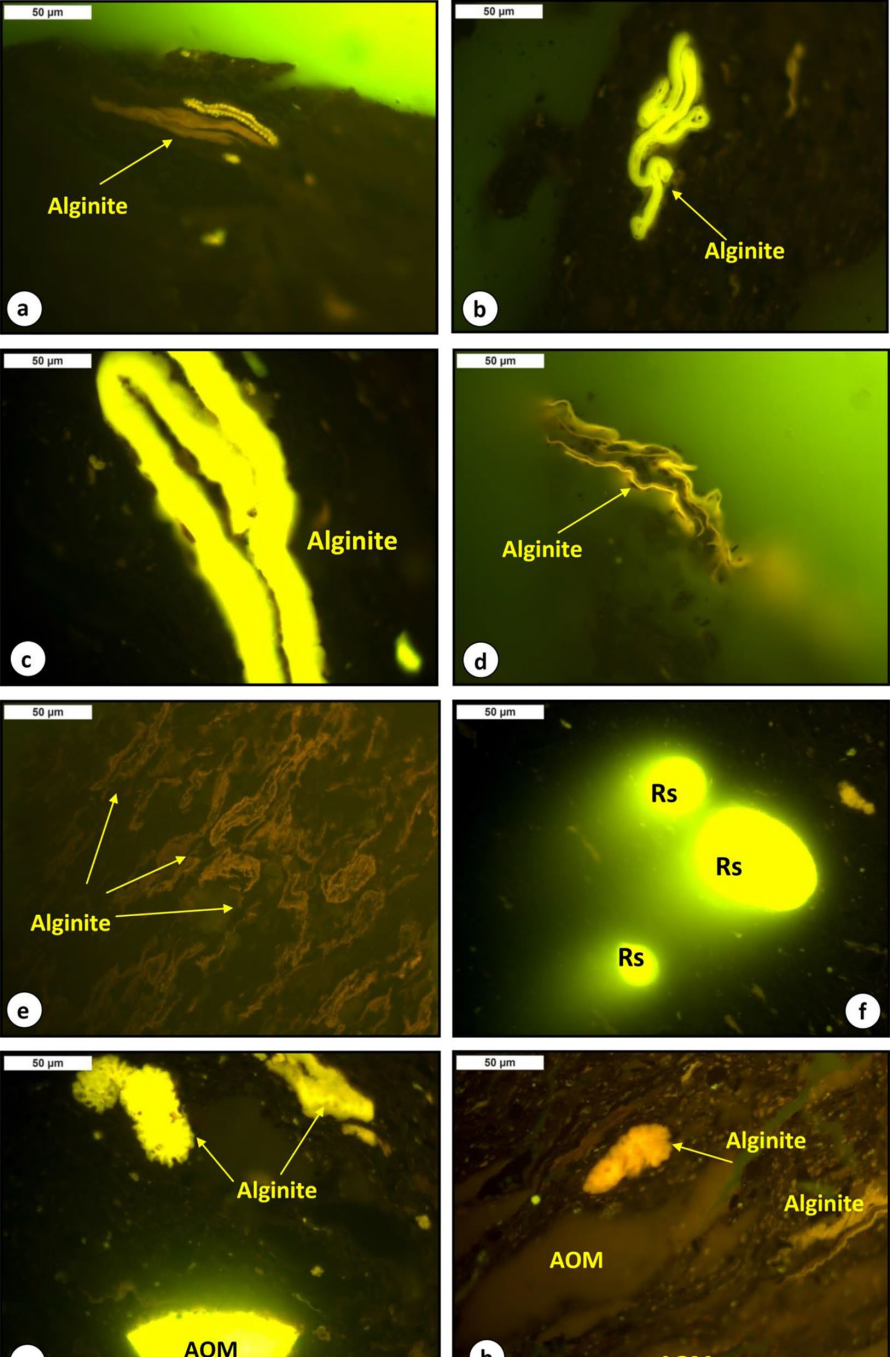
Photomicrographs of organic matter assemblage from the analyzed bituminous shales in the Paleocene Palana Formation; under UV light, field width = 0.2 mm: (**a**–**e**) yellow and brownish-yellow fluorescence alginite assemblages of mainly *telalginite* and *lamalginite*, (**f**) bright yellow fluorescing resinite (Rs), and (**g** and **h**) mixed organic matter of yellow and brownish-yellow fluorescence alginite assemblages of mainly *Botryococcus* and yellow and brownish-yellow fluorescence AOM.

Qualitative analysis of the organic matter assemblages in the analyzed shale samples was investigated using light microscopy under both plane-polarized reflected white light (Fig. 4) and ultraviolet (UV) light excitation (Fig. 5). The light microscopic analysis shows that the shale samples analyzed are made up of inorganic materials (mainly clay minerals), with organic matter dispersed in the mineral matrix (Fig. 4). The dispersed organic matter assemblages are mainly liptinitic materials and terrestrial organic matter of vitrinite and inertinite (Figs. 4 and 5).

UV light excitation has established the characteristics of the liptinitic material and is characterized by a green and yellow to brownish-yellow fluorescence (Fig. 5). The most commonly recognized liptinite in the analyzed shale samples was structured organic matter from yellow to greenish-yellow and brownish-yellow fluorescence alginite and was presented primarily as *telalginite* and *lamalginite* assemblies (Fig. 5a–c,e). The significant quantities of algal in the form of *telalginite* and *lamalginite* (Fig. 5a–c,e) indicate marine-reducing conditions during the deposition of the analyzed bituminous shales ^13–15^.

The occurrence of foraminifera assemblages in the studied samples (Fig. 4e) further suggests the marine setting. The *Botryococcus* alga was also frequently present in the analyzed samples and characterized by a yellow to brownish-yellow and orange fluorescence (Fig. 5g,h). Besides the telalginite and lamalginite assemblages, the presence of the *Botryococcus* algae indicates freshwater influence during deposition, and the analyzed bituminous shales were deposited in a reducing paralic environment. The other liptinitic materials such as resinite (Rs) and structureless amorphous organic matter (AOM) were also identified using UV light excitation, where distinct yellow to brownish-yellow fluorescence intensities were observed (Fig. 5f,h). However, the significant amounts of alginite assemblages and other liptinitic materials further suggest that the analyzed bituminous shales could be oil-shale resources.

In addition, the reflection measurements of the vitrinite phytoclasts were carried out on the 10 samples analyzed to assess the thermal maturity of the organic matter in the shale-bearing horizons. The reflectance of vitrinite (percent VRo) is generally valuable and a critical indicator commonly used to provide maturation information^16,17^. In this study, mean vitrinite reflectance values between 0.25 percent and 0.34 percent VRo (Table 1) for the organic matter intervals in the analyzed shale samples indicate immature organic matter.

**Table 1:** Qualitative chemical results of TOC content, Rock–Eval pyrolysis and ultimate analyses of the fourteen Palana bituminous shales and huminite/vitrinite reflectance (%VRo) results for representative ten bituminous shale samples in the Gurha mines at Bikaner, Rajasthan.

Mine	Horizons	Composite	Samples ID	Rock–Eval pyrolysis analysis									
				TOC wt.%	*S*_1_ (mg/g)	*S*_2_ (mg/g)	*S*_3_ (mg/g)	T_max_ (^o^C)	HI (mg/g)	OI (mg/g)	PY (mg/g)	PI (mg/g)	S_2_/S_3_ (mg/g)
Gurha	Upper horizon	GR-A	GR-1	18.23	0.93	88.01	2.80	428	483	15	88.94	0.01	31.43
			GR-2	25.26	1.59	136.52	3.55	430	540	14	138.11	0.01	38.46
			GR-3	30.30	2.83	148.04	5.57	426	489	18	150.87	0.02	26.58
		GR-B	GR-4	31.08	2.36	168.92	8.48	429	544	27	171.28	0.01	19.92
			GR-5	30.04	1.86	138.87	4.95	427	462	16	140.73	0.01	28.05
			GR-6	29.46	2.22	103.56	11.57	422	352	39	105.78	0.02	8.95
	Lower horizon	GR-C	GR-7	28.57	3.57	125.65	13.04	420	440	46	129.22	0.03	9.64
			GR-8	36.23	4.22	149.72	16.56	417	413	46	153.94	0.03	9.04
			GR-9	26.73	1.95	120.36	10.71	423	450	40	122.31	0.02	11.24
		GR-D	GR-10	32.09	3.96	154.40	11.38	423	481	35	158.36	0.03	13.57
			GR-11	28.64	2.26	122.44	12.29	421	428	43	124.70	0.02	9.96
			GR-12	33.04	2.79	152.05	15.43	421	460	47	154.84	0.02	9.85
		GR-E	GR-13	29.57	2.62	125.96	12.43	421	426	42	128.58	0.02	10.13
			GR-14	4.04	0.28	16.45	1.67	422	407	41	16.73	0.02	9.85

Mine	Horizons	Composite	Ultimate analysis							VRo (%)			
			C wt.%	H wt.%	S wt.%	N wt.%	O wt.%	Atomic ratios					
								H/C	O/C				
Gurha	Upper horizon	GR-A	78.60	7.60	2.18	2.61	9.02	1.16	0.09	0.29			
			83.83	8.30	1.70	2.27	3.91	1.30	0.03	0.25			
			64.40	6.13	1.84	2.26	25.37	1.14	0.30	0.25			
		GR-B	69.72	6.63	1.79	2.37	19.49	1.14	0.21	0.27			
			70.07	6.86	2.04	2.72	18.30	1.17	0.20	0.28			
			73.21	8.42	2.24	2.30	13.84	1.34	0.14	–			
	Lower horizon	GR-C	81.21	8.77	2.12	2.59	5.31	1.26	0.05	0.32			
			75.11	8.00	1.72	2.45	12.73	1.28	0.13	–			
			68.61	7.40	1.63	2.26	20.10	1.29	0.22	0.34			
		GR-D	76.20	8.06	1.55	2.27	11.92	1.27	0.12	–			
			71.31	7.72	1.85	2.42	16.70	1.30	0.18	0.33			
			66.86	7.51	1.74	2.18	21.72	1.35	0.24	–			
		GR-E	81.58	8.68	2.09	2.76	4.88	1.28	0.04	0.31			
			79.23	8.72	1.91	2.74	7.40	1.32	0.07	0.33			

TOC, Total organic Carbon, wt.%; *S*_1,_ Volatile hydrocarbon (HC) content, mg HC/ g rock; *S*_2,_ Remaining HC generative potential, mg HC/ g rock; *S*_3_, carbon dioxide content, mg CO_2_/g rock; T_max,_ Temperature at maximum of *S*_2_ peak; OI, Oxygen Index = *S*_3_ × 100/TOC, mg CO2/g TOC; HI, Hydrogen Index = *S*_2_ × 100 / TOC, mg HC/ g TOC; PY, Potential Yield = *S*_1_ + *S*_2_ (mg/g); PI, Production Index = *S*_1_/(*S*_1_ + *S*_2_); C, Carbon, wt.%; H, Hydrogen, wt.%; S: Sulphur, wt.%, ; N, Nitrogen, wt.%; O, Oxygen, wt.%.

### TOC and sulfur contents

Table 1 lists the results of the TOC and sulfur contents of the analyzed shale samples and allows assessing the origin and or state of the environmental conditions^18,19^. The TOC wt.% content has been commonly used to provide information on the amount of organic matter regarding the richness of organic matter during deposition^20,21^. As expected, the analyzed bituminous shale samples have excessively high organic carbon content in the range of 18.23–36.23 wt% (Table 1), except for one sample with a relatively low TOC of 4.04wt%. The relatively low TOC of 4.04% can be attributed to the diluted organic matter because of relatively terrestrial detrital inputs^22,23^. However, the high TOC contents suggest the enrichment of organic matter (OM) during deposition time of the analyzed samples. The primary mechanism controlling OM enrichment is preservation of the organic matter under reducing environmental conditions during the deposition of these bituminous shale sediments. The presence of reducing environmental conditions would likely have caused high growth of aquatic-derived organic matter (i.e., phytoplankton algae)^24^.

The common phytoplankton algae recorded in the bituminous shale samples are mainly *telalginite* and *lamalginite*, with *Botryococcus* algae as shown in Fig. 5. The abundance of these algae-derived constituents can be interpreted as an indicator of a marine-reducing condition during the deposition^13–15,25^. The presence of the phytoplankton algae deposited under reducing environmental conditions was further established from the acyclic isoprenoid distributions [i.e., pristane (Pr) and phytane (Ph)] as presented in the next subsection.

**Figure 6:**
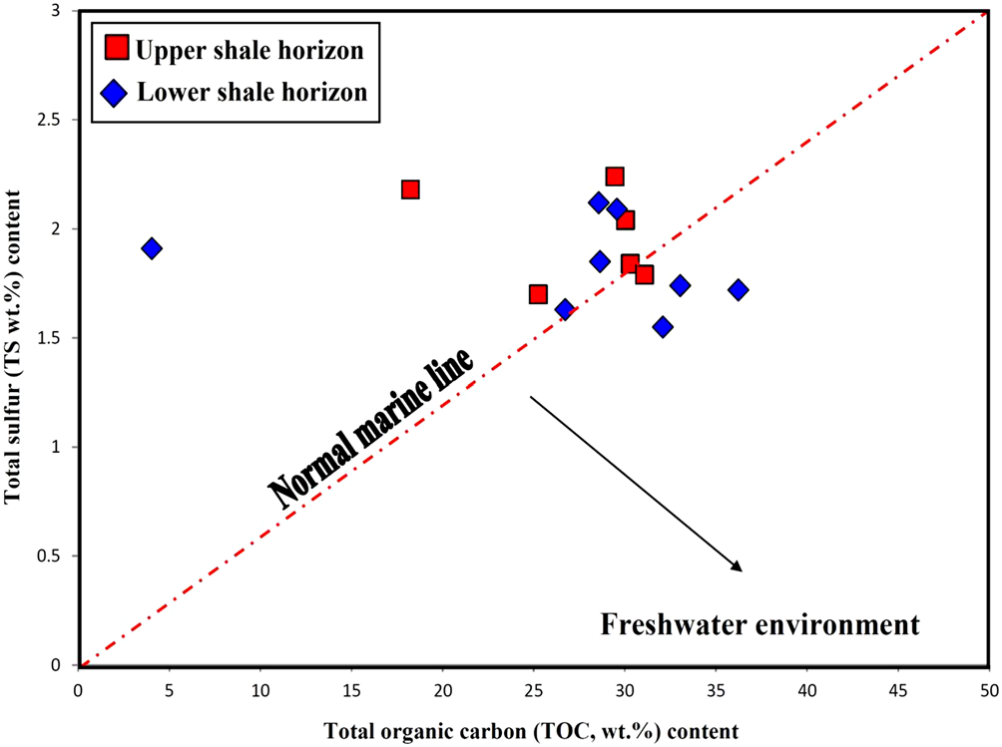
Cross-plot of the sulfur content plotted against TOC (wt%), suggesting a marine environment, with little connection to the freshwater (Paralic setting) during deposition of the analyzed Palana shales (modified after Berner and Raiswell^18^).

In this study, TS, wt% content in the analyzed shale samples was also measured (Table 1). The content of TS in the analyzed samples ranged from 1.46 wt% to 2.39 wt.% (Table 1), with an arithmetic mean of 1.94 wt.%. As the principal source of high sulfur of more than 2 indicates marine water under reducing conditions^15^, and low sulfur content (TS < 0.5 wt%) indicates non-marine sediments (freshwater)^18,26^ . Therefore, the sulfur content in this study implies that the analyzed shales were mainly deposited in a marine environment, with little connection to freshwater. The interpretation of the paralic environment condition is supported by the variety of low and high TS content values (Table 1), and corresponds with the correlation between TOC and TS contents (Fig. 6).

### Geochemical Kerogen characteristics

Quantitative and qualitative pyrolysis was investigated throughout this study and all the different pyrolysis parameters and pyrolysates of the analyzed bituminous shales were measured and identified (Tables 1 and 2). From these measurements, we can assess the kerogen types and their characteristics and compositions, representing the source of organic matter input in the analyzed shale samples.

**Table 2:** Quantitative chemical results of extracted shale samples in the Gurha mines at Bikaner, Rajasthan, including some compounds calculated from Py–GC pyrograms and mass fragmentogram of m/z 85 ion in GC–MS of the saturated hydrocarbon.

Mine	Horizons	Composite	Samples ID	*n*-alkane and isoprenoids				Pyrolysis–gas chromatography (Py-GC)					
								Kerogen Type			Petroleum Compositions		
				Pr/Ph	Pr/C_17_	Ph/C_18_	CPI	2-, 3-dimethylthiophene	Ortho-xylene	*n*-C_9_	C_1_-C_5_ (%)	C_6_-C_14_ (%)	+ C_15_ (%)
Gurha	Upper horizon	GR-A	GR-1	1.05	0.12	0.29	2.10	14.61	20.22	65.17	23.78	39.42	36.80
			GR-2	–	–	–	–	10.28	25.30	64.43	21.02	40.24	38.74
			GR-3	–	–	–	–	11.11	24.28	64.61	21.64	39.52	38.84
		GR-B	GR-4	0.82	0.17	0.35	1.97	10.81	26.35	62.84	21.32	40.72	37.95
			GR-5	–	–	–	–	12.93	21.77	65.31	20.75	39.07	40.18
			GR-6	–	–	–	–	10.22	22.58	67.20	26.72	32.33	40.95
	Lower horizon	GR-C	GR-7	0.76	0.12	0.27	2.03	14.98	20.29	64.73	25.40	39.10	35.50
			GR-8	–	–	–	–	13.51	18.92	67.57	22.75	38.07	39.18
			GR-9	1.18	0.11	0.31	2.02	15.15	20.71	64.14	24.72	34.33	40.95
		GR-D	GR-10	–	–	–	–	12.07	26.55	61.38	25.40	38.10	36.50
			GR-11	1.35	0.15	0.30	2.08	10.34	24.14	65.52	25.48	38.42	36.10
			GR-12	–	–	–	–	13.13	34.06	52.81	23.02	38.24	38.74
		GR-E	GR-13	1.00	0.15	0.28	1.99	11.16	23.51	65.34	21.98	39.32	38.70
			GR-14	–	–	–	–	12.13	32.06	55.81	22.72	39.72	37.55

Pr, Pristane; Ph, Phytane; CPI, Carbon preference index (1): {2(C_23_ + C_25_ + C_27_ + C_29_) / (C_22_ + 2[C_24_ + C_26_ + C_28_] + C_30_)} 2,3 Dimeth. (%) – percent concentration of 2,3 dimethylthiopene in relation to O-xylene and n-C9; O-xylene (%), percent concentration of O-xylene in relation to 2,3 dimethylthiopene and n-C9; n-C9 (%), percent concentration of n-C9 in relation to 2,3 dimethylthiopene and O-xylene; n-C1–n-C5 (%), percent concentration of n-C1–n-C5 in relation to n-C6–n-C14 and n-C15 + ; n-C6–n-C14 (%), percent concentration of n-C6–n-C14 in relation to n-C1–n-C5 and n-C15 + ; n-C15 + (%), percent concentration of n-C15 + in relation to n-C1–n-C5 and n-C6–n-C14.

**Figure 7:**
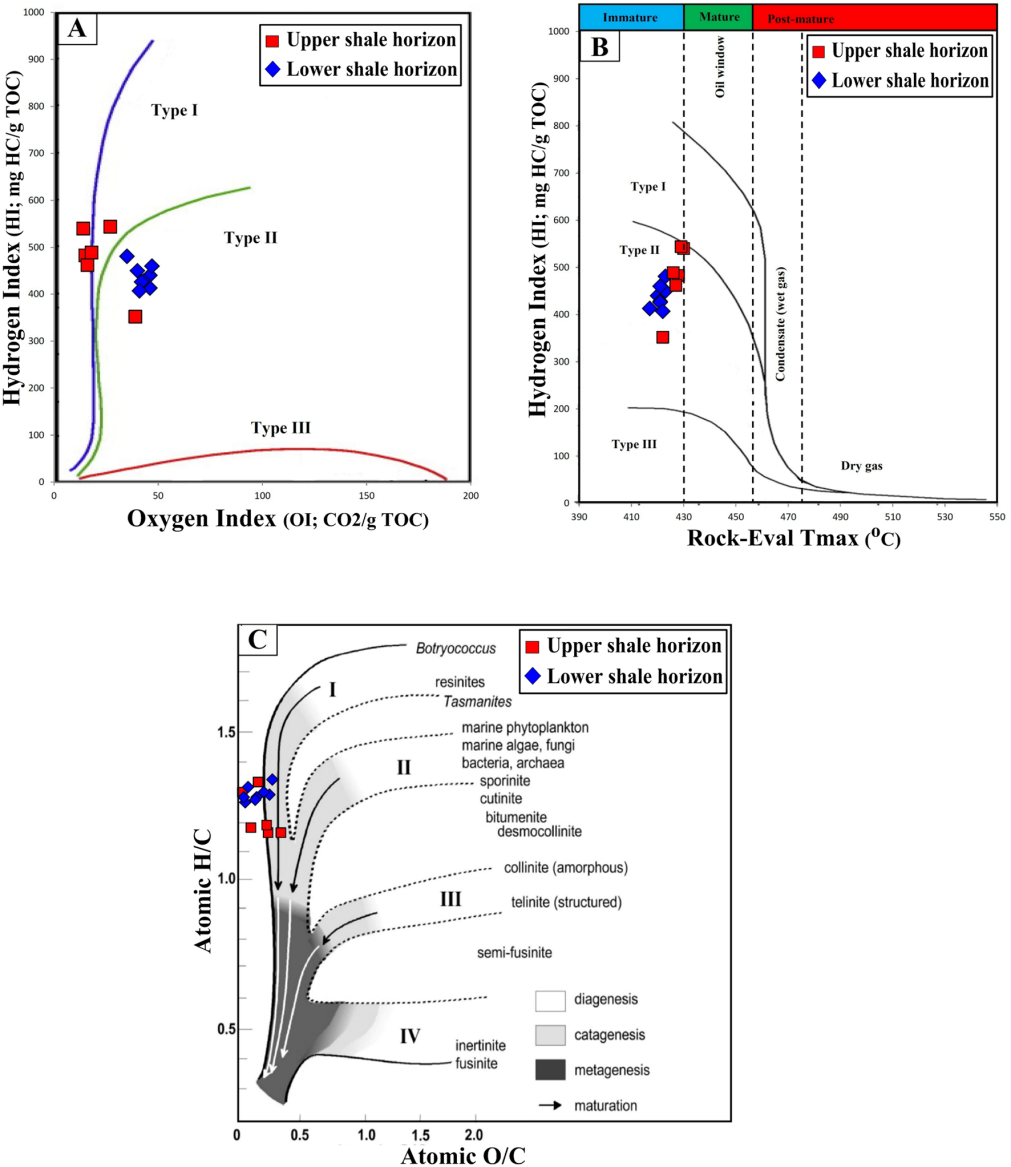
Geochemical correlations between Rock–Eval hydrogen index (HI), oxygen index (OI), and *T*_max_, and hydrogen-to-carbon (H/C) versus oxygen-to-carbon (O/C) ratios, showing that the analyzed shale samples are dominated by Type II kerogen (adopted after Peters and Cassa^20^).

The qualitative kerogen type in the analyzed shale samples was evaluated based on their geochemical parameters of Rock–Eval HI and OI^20,21,27,28^. In general, the analyzed shale samples are dominated by the HI with values between 352 and 544 mg HC/g TOC, and low values of OI parameters in the range of 14 and 47 mg CO_2_/g TOC (Table 1). Based on the overall Rock–Eval results, the analyzed shale samples mainly fell into Type II kerogen, as obtained from the Van Krevelen diagram of HI against OI of samples (Fig. 7a). A modified-HI versus *T*_max_ plot of the analyzed samples correlates well with the interpretation based on the Van Krevelen diagram and further confirmed the presence of Type II kerogen in the immature zone (Fig. 7b).

However, other chemical qualitative analyses of the ultimate elemental (CHNSO) are used to corroborate the kerogen characteristics^21,29^. The results of CHNSO elements were used to calculate the ratios of hydrogen-to-carbon (H/C) and oxygen-to-carbon (O/C) atomics (Table 1) and provided useful results on the kerogen facies in the analyzed samples. The H/C and O/C atomic ratios were calculated in the range of 1.14–1.38 and 0.09–0.35, respectively (Table 1). According to Peters and Cassa^20^ and Hunt^21^, the high ratios of H/C atomic indicate rich oil-prone Type I and II kerogens, while high O/C atomic indicates gas-prone Type III kerogen. Given this, the kerogen type in the analyzed shale samples is mainly classified as Type II, according to Van Krevelen’s diagram of H/C and atomic ratios (Fig. 7c). This correlates well with the kerogen characteristics derived from the Rock–Eval HI and OI results (Fig. 7a,b).

Another useful geochemical application for obtaining the composition of the thermally decomposed kerogen is the quantitative pyrolysis of open pyrolysis (Py-GC), and provides accurate and more reliable assessments of the kerogen type^30,31^. The Py-GC is provided for the pyrolysate *S*_2_ of the number of hydrocarbons generated by the thermal cracking of kerogen and used to deduce the nature of kerogen^32,33^.

**Figure 8:**
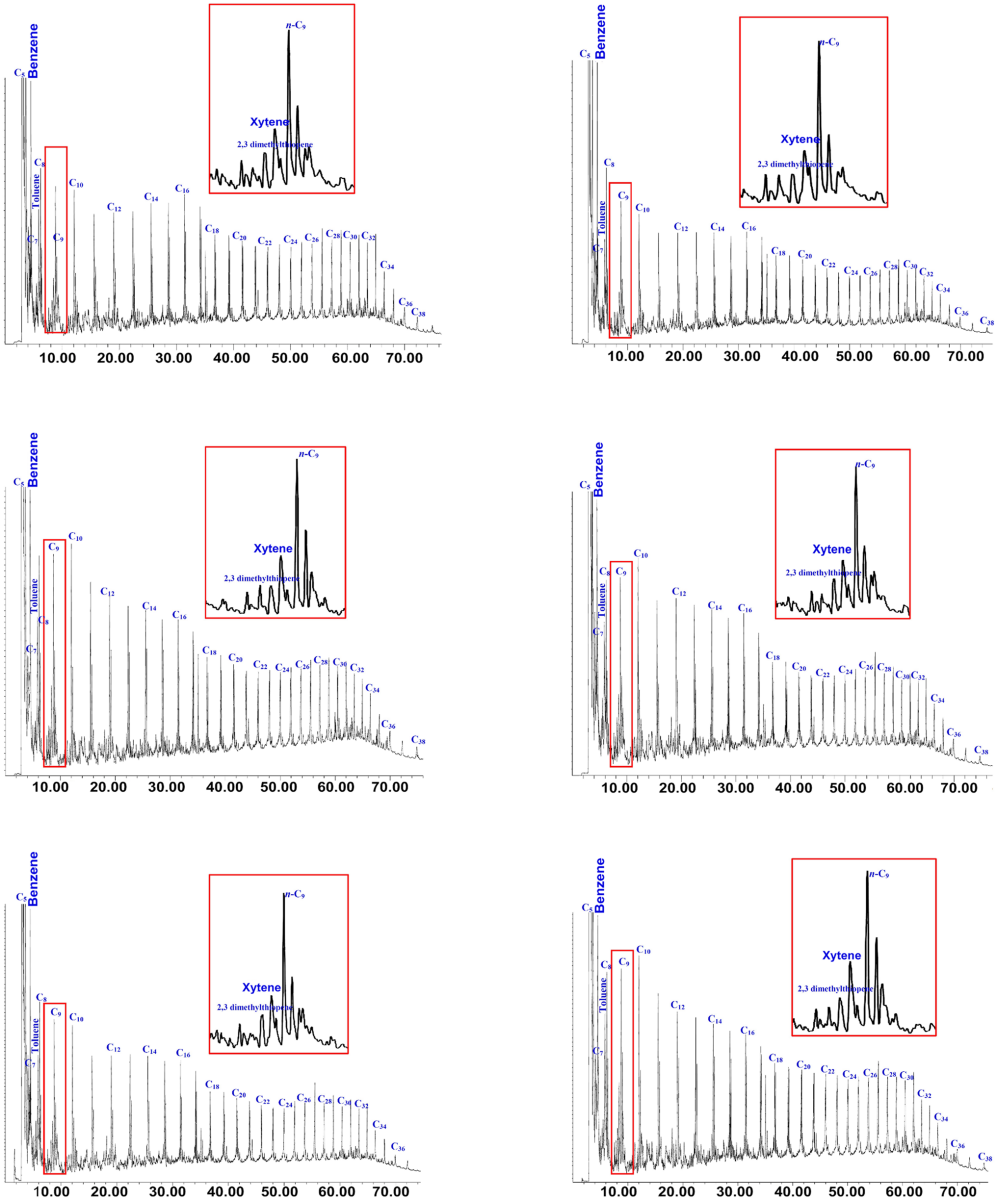
Pyrolysis GC pyrograms of selected analyzed Palana shale samples, showing labeled peaks of *n*-alkene/alkane doublets, sulfur-lean organic matter, and aromatic hydrocarbons (toluene, xylene, and benzene) used as kerogen and petroleum type proxies (see Fig. 9).

Figure 8 shows the chromatograms of the pyrolysate *S*_2_ material of the analyzed shale samples. The chromatograms of the analyzed samples display a bimodal distribution of methane peaks followed by predominantly *n*-alkene/*n*-alkane doublets of up to *n*-C_30_ (Fig. 8). The pyrolysates of the analyzed samples also contain minor amounts of organic sulfur, and light aromatic hydrocarbons of toluene, xylene, and benzene (Fig. 8). This distribution of abundant *n*-alkene/*n*-alkane doublets and relatively low light aromatic hydrocarbons is compatible with the presence of oil-prone kerogen II^34^.

**Figure 9:**
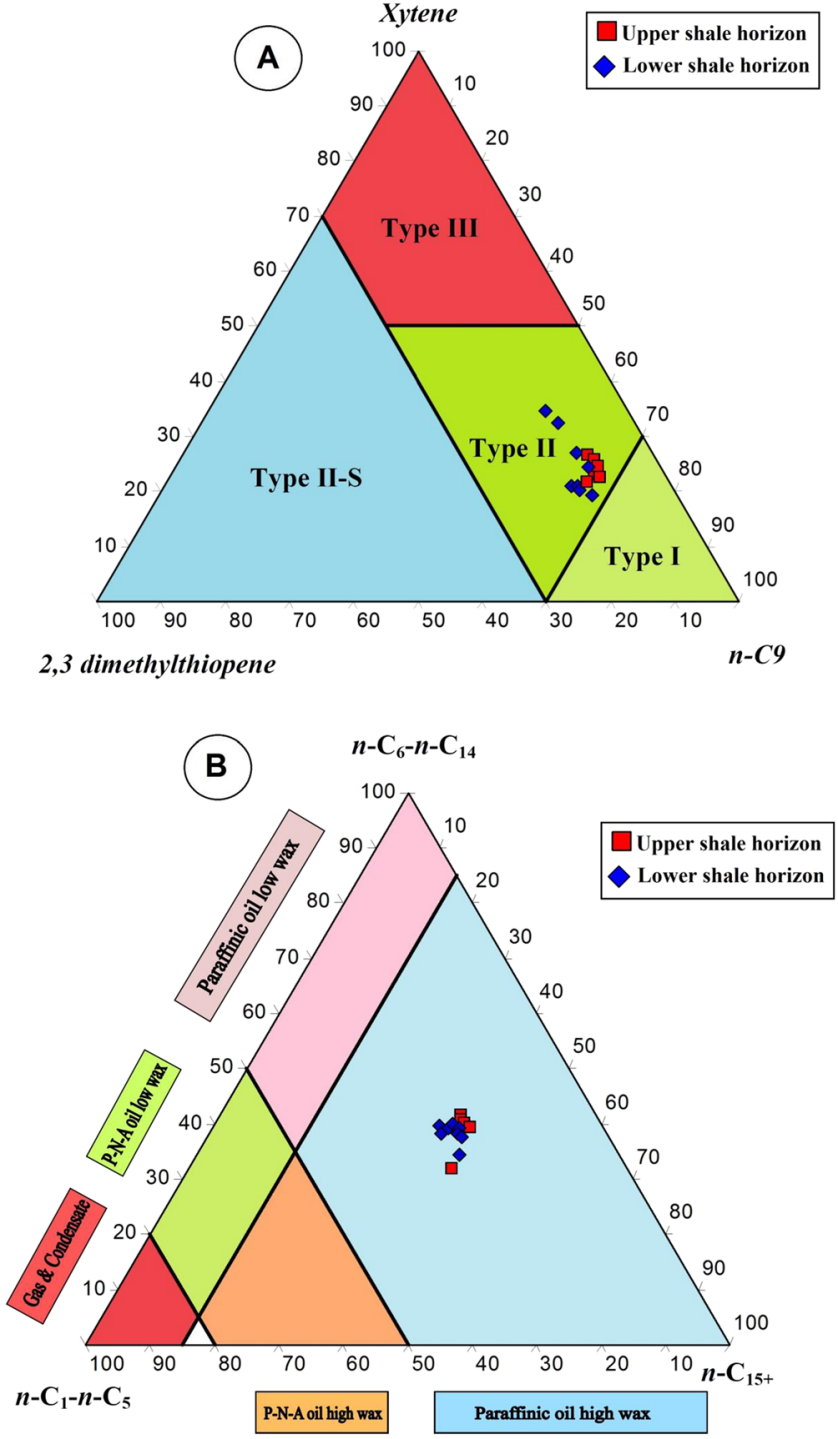
Ternary diagrams on the kerogen and petroleum type proxies derived from Py-GC data (**A**) distinguish the type of organic matter using relative percentage of compounds 2,3-dimethylthiophene, O-xylene (1,2- dimethylbenzene) and n-non-1-ene (n-C9:1), showing Type II kerogen (adapted after Eglinton et al.^36^), and (**B**) ternary diagram after Horsfield^30^ based on n-alkyl chain length distribution, showing that the analyzed shale samples are expected to generate a paraffinic high-wax oils. Tridraw 4.5 software is used to create this figure, https://tridraw.software.informer.com/4.5/.

This inference of the kerogen characteristics is further supported by the specific distributions of the pyrolysate distributes of 2-, 3-dimethylthiophene, ortho-xylene, and *n*-non-1-ene components^35^. The pyrolysate distributes of 2-, 3-dimethylthiophene, ortho-xylene, and n-non-1-ene of the analyzed samples (Fig. 8 and Table 2) were plotted on Eglinton et al.^36^ ternary diagram, giving rise to kerogen Type II (Fig. 9a). The distributions of the pyrolysate *S*_2_ material derived from the Py-GC chromatograms (Fig. 8) are further used to classify the composition of the petroleum-generating facies in the analyzed samples by applying specific pyrolysate distributions of total resolved C_1_ to C_5_, and the sum of the *n*-alkenes/*n*-alkanes in the C_6_ to C_14_, and C_+15_ (Table 2), and plotting it on the Horsfield^30^ ternary diagram. On this ternary diagram, the analyzed bituminous shale samples are plotted in the field of high-wax paraffinic oils (Fig. 9b).

The derived organic facies are compatible with a useful optical application for obtaining the quantitative organic matter type under microscope, and provides accurate and more reliable assessments of the type of organic facies in the analyzed bituminous shale samples. The kerogen microscopic results, including the organic facies in the analyzed samples were dominated by primarily aquatic-derived organic matter (i.e., phytoplankton algae) with minor amounts of vitrinite and inertinite organic matter that derived plants (Figs. 4 and 5). However, the predominantly Type II organic facies in the analyzed shales (Figs. 7 and 9A) is consistent with the presence of high contributions of phytoplankton algae of marine origin, i.e., *telalginite* and *lamalginite* (Fig. 5a–e), and low contributions of *Botryococcus* algae (Fig. 5g,h) of the lacustrine environment.

### Normal alkane and isoprenoid distributions

**Figure 10:**
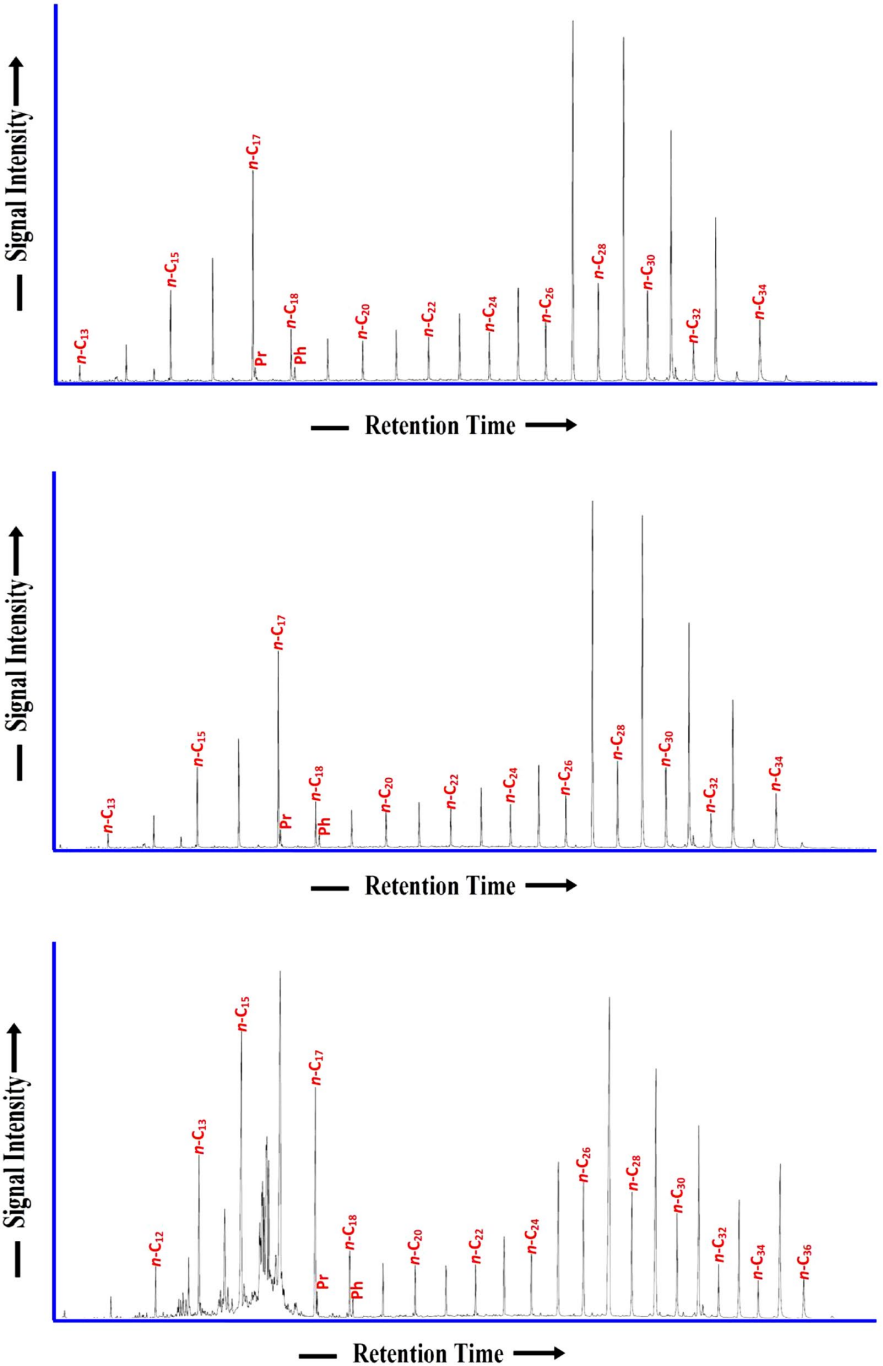
*n*-alkane and isoprenoid distribution according to m/z 85 of the selected studied shale samples.

The lipid biomarkers of the saturated hydrocarbon in the selected six bituminous shale samples were detected as the normal alkane and isoprenoid distributions in the chromatograms (Fig. 10). The chromatograms showed a bimodal distribution of normal alkanes between C_14_ and C_33_, with an excess of middle-chain (C_25_–C_29_) *n*-alkanes (Fig. 10). This bimodal distribution of lipid biomarkers implies a mixture of organic matter with aquatic organic matter and many land plants. The occurrence of carbon numbers ranging from *n*-C_14_ to *n*-C_27_ suggests inputs from aquatic organic matter, where odd carbon numbers of more than C_27_ and maximizing at *n*-C_29_ indicates the input of terrestrial higher plants^37,38^. The presence of a significant number of land plant inputs suggests a relatively high carbon preference index in the range of 1.97 and 2.10 (Table 2).

**Figure 11:**
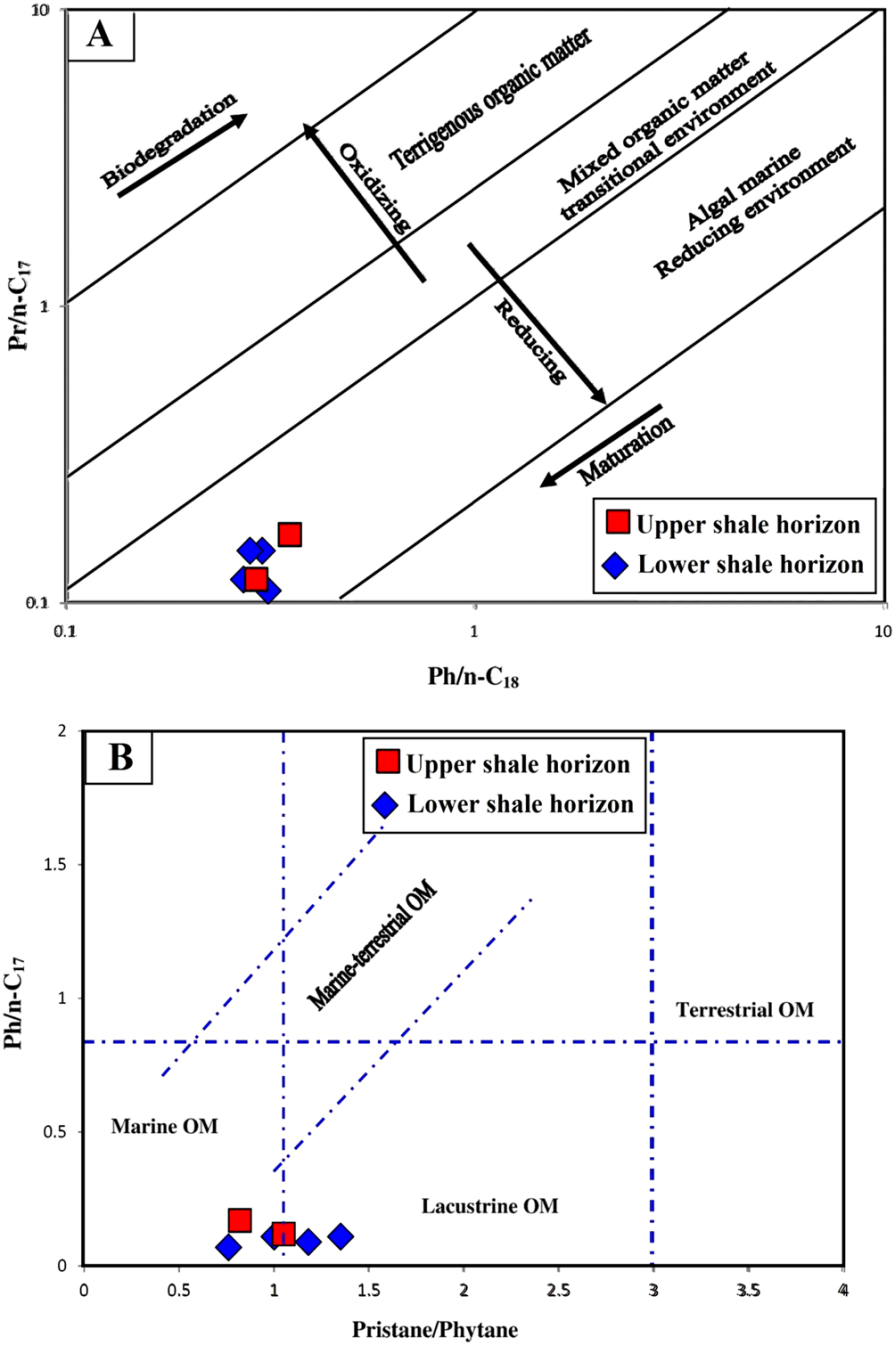
Biomarker cross-plots of isoprenoid ratios: Pr/Ph, Pr/C_17_, and Ph/C_18_, showing that the analyzed shale samples received a high contribution of algal organic matter and deposited in mainly marine origin and little connection to freshwater and preserved under reducing environmental conditions.

The acyclic isoprenoid hydrocarbons, i.e., pristane (Pr) and phytane (Ph), were also recorded in the chromatograms of the samples (Fig. 10). Such acyclic isoprenoids are the most commonly used geochemical biomarkers of the redox conditions and inputs of organic matter in depositional environments^39,40^. Specifically, the Pr and Ph concentration occur in almost equal amounts and its Pr/Ph ratio ranged from 0.82 to 1.05 (Table 2). The isoprenoid ratios relative to the *n*-alkane concentrations (C_17_–C_18_) were also calculated, yielding Pr/*n*-C_17_ and Ph/*n*-C_18_ ranges of 0.22–0.44 and 0.71–0.97, respectively (Table 2). The acyclic isoprenoid distributions (Fig. 10), with relatively low ratios of Pr/Ph, pristane/*n*-C_17_, and phytane/*n*-C_18_, suggest that the analyzed shale samples received a high contribution of algal organic matter preserved under reducing conditions (Fig. 11a), of mainly marine origin and little connection to a freshwater (lacustrine) environment (Fig. 11b). This finding is further confirmed by the dominant presence of phytoplankton algae of marine origin, i.e., *telalginite* and *lamalginite* (Fig. 5a–e), with low contributions of *Botryococcus* algae (Fig. 5g,h) of the lacustrine environment.

## Discussion

### Origin of organic matter and environmental conditions

The sources of organic matter and its environmental conditions during the deposition of the bituminous bearing shale horizons in the Palana Formation were assessed from integrated geochemical and petrographic data. This was coupled with the lipid biomarker distributions of normal alkane and isoprenoid in the saturated hydrocarbon, as previously described. The geochemical results show that the analyzed shale samples are originally rich and dominated by Type II kerogen (Fig. 7). An abundance of this kerogen type indicates the dominance of aquatic-derived organic matters, such as algal and bacteria. The dominance of Type II kerogens correlates well with kerogen assemblages observed under the microscope and is consistent with the predominance of liptinitic materials, including alginite of the *telalginite*, *lamalginite* and *Botryococcus* assemblages, resinite and AOM (Fig. 5). These organic matter constituents were previously named as sapropel or sapropelite^28^, and commonly deposited under reducing conditions^13,15,41^. The *telalginite* and *lamalginite* assemblages are typical phytoplankton algae of marine origin^15,41,42^, while the *Botryococcus* algae are indicative of lacustrine environments^43^. Therefore, the dominant presence of *telalginite* and *lamalginite* with a low continuation of freshwater algae, i.e., *Botryococcus* (Fig. 5) suggests a mainly marine environment with freshwater influence (paralic setting) during the deposition of the Palana bituminous shale sediments.

The large quantities of organic matter derived from phytoplankton algae indicate both reducing environment and warm climatic conditions^15,25^ enhancing the abundance of primary nutrients in seawater and, subsequently, increased the biological productivity of algae and other microorganisms. The distribution of the isoprenoids and their ratios (Pr/Ph Pr/*n*-C_17_, and Ph/*n*-C_18_) further proves the high concentration of marine alga and reducing environmental conditions during deposition of the Palana bituminous shale sediments (Fig. 11). These reducing environmental and warm climatic conditions during the sedimentation of the shale-bearing horizons in the Palana Formations, contribute to the preservation and high bioproductivity that consequently enhanced the enrichment of organic matter, as indicated by high TOC values of up to 36.23 wt. % (Table 1).

### Contribution to unconventional oil-shale resources

The properties of the organic-rich shale sediments and their contribution to petroleum-shale resources are generally assessed based on organic type and thermal ripening temperatures^20,21,28,41,44,45^.

**Figure 12:**
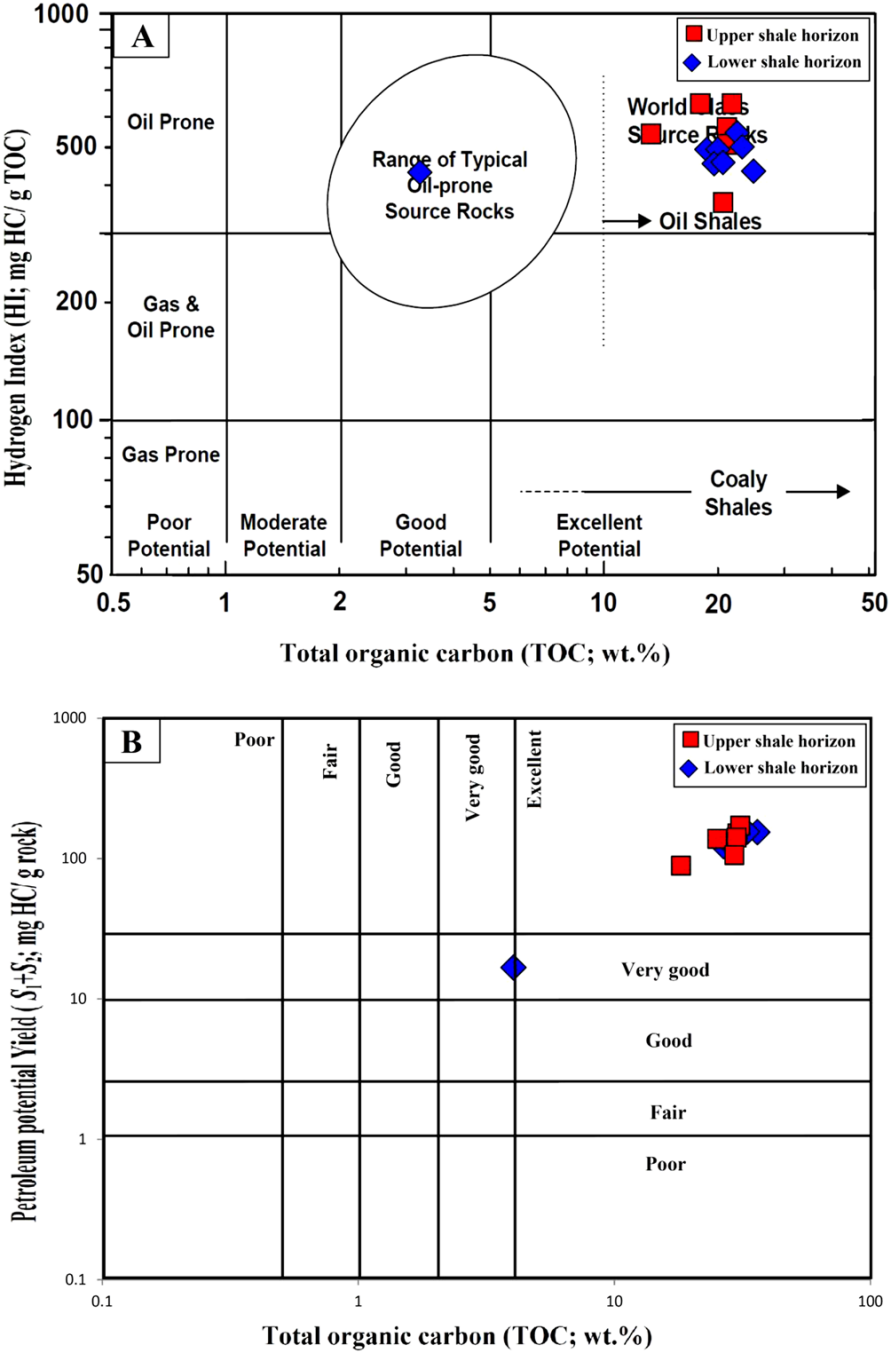
Geochemical correlations between TOC content and Rock–Eval data, (**A**) plot of TOC vs. HI and (**B** ) plot of TOC vs. PY(S1 + S2). It implies that the shale horizons of the Palana Formation in the Bikaner sub-basin are oil-shale resources and should generate significant amounts of oil in the sufficient thermal maturation.

In this study, the integrated geochemical results and the kerogen microscopic features were discussed to characterize the organic facies and their potential as an oil-shale resource in the bituminous shale-bearing horizons of the Palana Formation in the Bikaner sub-basin of Rajasthan state. These integrated findings showed that the analyzed shale samples from different shale-bearing horizons are similar in the characteristics of the organic facies and show mainly Type II kerogen. This is attributed to the source of organic matter inputs and their depositional environment conditions. The analyzed samples from both shale-bearing horizons were primarily derived from aquatic organic matter, with high contributions of algal masses (Fig. 5), depositing in reducing environmental conditions. These are common in oil-shale deposits and have hydrogen-rich Type II kerogen, as demonstrated from their HI and H/C values exceeding 352 mg HC/g TOC and 1.10%, respectively (Fig. 7). These chemical results are consistent with the pyrolysate distributions of *S*_2_ derived from the Py-GC analysis (Fig. 8), indicating that the analyzed shale samples contain mainly oil-prone Type II kerogen (Fig. 9a). Therefore, the analyzed samples in both shale horizons of the Palana Formation in the Bikaner sub-basin are considered as an oil-shale resource (Figs. 9b; and 12a), and should generate significant amounts of oil in sufficient thermal maturation, as indicated from high TOC content and pyrolysis (*S*_1_ + *S*_2_) yields exceeding 18% and 100 mg HC/g rock, respectively (Fig. 12b).

Besides, the oil generation capacity of the analyzed oil-shale was further assessed from the thermal maturation of the organic matter. The thermal maturity level of the analyzed shale samples was evaluated using both optical and chemical maturity indicators. The most accurate maturity indicator was the optical vitrinite reflectance (%VRo), providing valuable information about the organic maturation and evolution of the petroleum generation capacity^16,46^. The VR values ranged from 0.25 to 0.34% (Table 1), confirming that the analyzed oil-shale samples are still in an immature stage of the oil-generation window.

**Figure 13:**
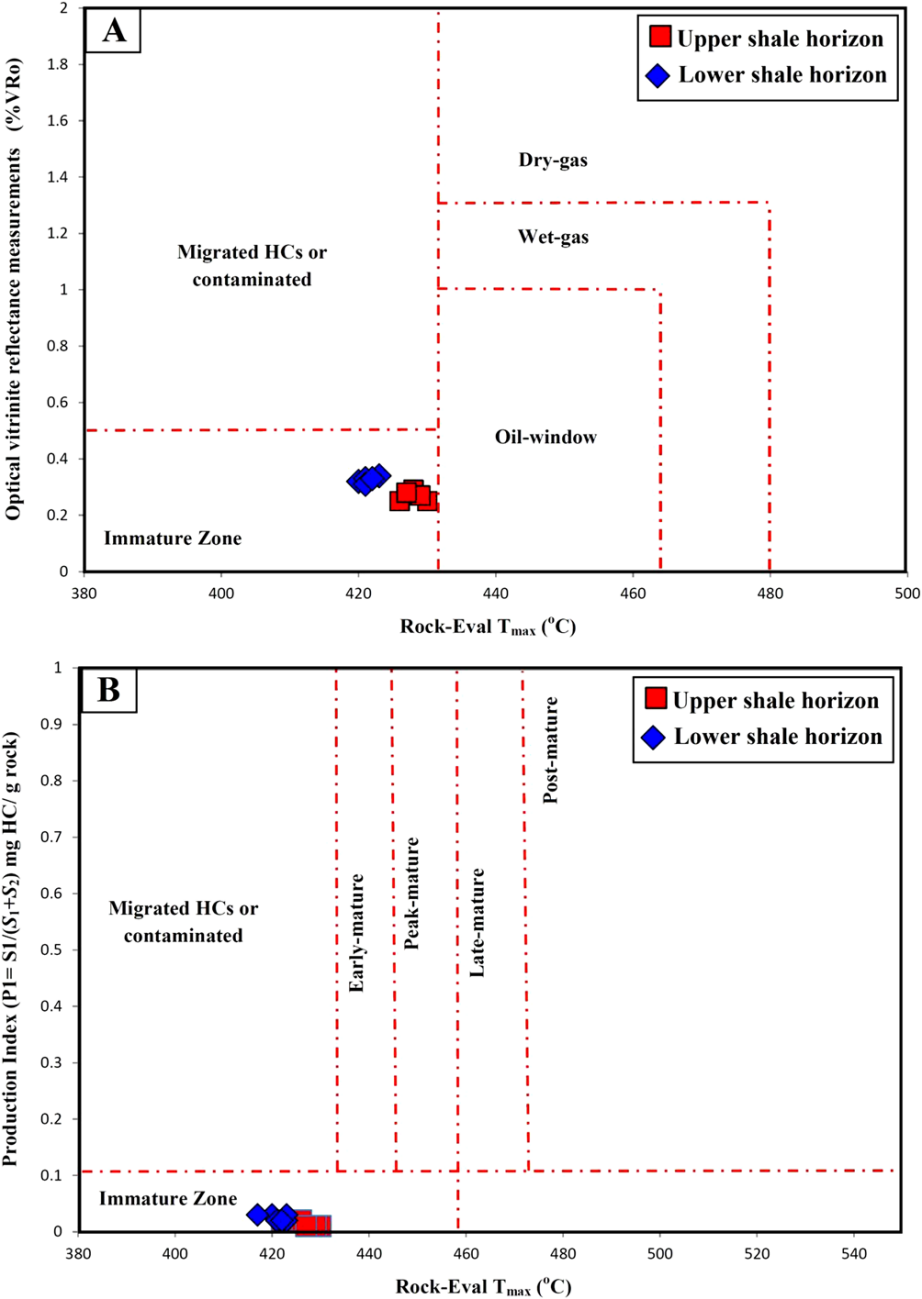
Geochemical cross-plots of (**a**) Pyrolysis T_max_ versus measured vitrinite reflectance (%VRo) and (**b**) Pyrolysis T_max_ versus production index (PI), showing that the analyzed shale samples are still at an immature stage for oil-generation.

The temperature of the maximum pyrolysis rate (*T*_max_) is also commonly used to estimate the maturity of kerogens; as they mature, their *T*_max_ values increased^28,47,48^. During the pyrolysis analysis, the *T*_max_ of the analyzed samples was determined as 417–430 °C (Table 1), confirming immature organic matter (Fig. 13a). Subsequently, the level of thermal maturation was evaluated using the PI of the analyzed samples^20,21^. The PI values were below 0.05 (Table 1) because of the presence of immature organic matter and consistent with the optical vitrinite reflections and chemical T_max_ values (Fig. 13b). Therefore, the bituminous shales (oil-shale) in this study have not yet reached the oil window maturity to generate significant quantities of oil. Given this, the low maturity bituminous shales of the Palana Formation in the Bikaner sub-basin of Rajasthan state are likely unconventional oil-shale resources and could be artificially pyrolyzed to release significant amounts of oil using unconventional production techniques.

## Data and methods

In this study, fourteen high bituminous shales of the Paleocene Palana Formation were collected from the working faces of the Gurha lignite mines in Bikaner, Rajasthan (Fig. 1). Shale samples were collected from both the lower and upper shale horizons (Fig. 3). Several precautions were made throughout the sampling to construct a seam profile in the laboratory including pillar sampling. Geochemical and organic petrographic characteristics on the high bituminous shale horizons were performed using TOC content, Rock–Eval pyrolysis ultimate (CHNS), bitumen extraction, gas chromatography-mass spectrometry (GC–MS), kerogen microscopy, and vitrinite reflectance. The shale samples were pulverized to lower than 200 mesh size and subjected to various organic geochemical analyses, namely TOC content and bulk pyrolysis, ultimate, and bitumen extraction.

The analyzed samples were examined using a Rock–Eval 6 pyrolysis machine and a TOC component by the procedure of Espitalié et al.^27^ and Espitalie, et al.^49^ . The parameters *S*_1_ (amount of free hydrocarbons), *S*_2_ (amount of hydrocarbons generated by thermal cracking of non-volatile organics), *S*_3_ (amount of CO_2_ generated by kerogen pyrolysis), *T*_max_ (temperature of highest hydrocarbon amount released by kerogen cracking) of the samples, and the TOC were measured during pyrolysis. The geochemical parameters of hydrogen index (HI = *S*_2_*100/TOC), oxygen index (OI = *S*_3_*100/TOC), potential yield (PY = *S*_1_ + *S*_2_), and production index (PI = *S*_1_/*S*_1_ + *S*_2_) were estimated by Peters and Cassa (1994) (see Table 1)^20^.

The ultimate analysis was performed on the analyzed shale samples following as per of ASTM D5373-08 (1993) guidelines^50^. The fine grounded whole samples were analyzed using an elemental analyzer to measure CHNS contents in percentage. Subsequently, the oxygen content was calculated based on the difference in the percentages of the CHNS contents. The atomic ratios of H/C and O/C were then calculated following the procedure of ASTM D5373-08 (1993)^50^.

Furthermore, bitumen (*S*_1_) was extracted in the Soxhlet apparatus using an azeotropic mixture of dichloromethane (DCM) with methanol (CH_3_OH) with a ratio of 93:7 for approximately 72 h. The extracted rock samples of 1–2 mg were subjected to pyrolysis gas chromatography (Py-GC). The pyrolysis method was performed on the extracted samples by heat using the Frontier Lab Pyrolyser System. The Py-GC analysis was programmed from 40 to 300 °C before pyrolysate flowed through the GC column between 300 and 600 °C. The chromatograph of the pyrolysates at 600 °C shows methane peaks, followed by a series of *n*-alkene/*n*-alkane doublets, with aromatic components. They were used to identify their *S*_2_ composition and the structural characteristics of kerogen.

The saturated fraction from the extracted bitumen in the six representative samples was then collected by using a liquid column chromatography separation method using a petroleum ether solvent with silica gel topped with alumina oxide. The saturated fraction was analyzed by applying the Agilent 5975B inert MSD gas chromatography-mass spectrometry (GC–MS). An HP-5MS column was attached and the temperature was controlled from 40 to 300 °C at a rate of 4 ºC/min with a 30 min hold at a temperature of 300 ºC. Consequently, lipid biomarkers in the saturated hydrocarbon such as *n*-alkanes and isoprenoids were produced using an ion chromatogram of m/z 85, and their ratios were measured from their peak heights.

Besides the geochemical analyses, the organic petrographic analysis was conducted on 10 shale samples using the polished block technique. The tests include kerogen microscopy and reflectance measurements of the vitrinite organic matter.

The whole-rock samples were approximately crushed to around pea-sized fragments (2–3 mm) and inserted into molds using a combination of Serifix-resin and hardener cold mounting. The block samples were further slowly ground using various size silicon carbide papers and eventually polished with powder-deagglomerate alumina and OP-suspension solution, respectively. Organic matter characteristics were analyzed using oil immersion under a plane-polarized reflected light with a Leica DM 2700P microscope and assisted with an fitted MSP 200 coal photometry device and fluorescence attachment.

The vitrinite reflectance (%VRo) of the samples was optically analyzed under a plane-polarized reflected light using standard methods, as described by Taylor et al.^13^. The reflectance was calibrated using a spinal standard with a 0.42% reflectance value, and the values reported were arithmetic means from 25 measurements per sample.

## Conclusions

Geochemical and organic petrographic investigations were performed on high bituminous shale-bearing horizons within the Paleocene Palana Formation in the Gurha mine, NW India, and used to assess the origin of organic matter and their connection to the oil-shale as an alternative unconventional energy resource. The main conclusions are outlined below.

1. The geochemical findings indicate that the analyzed shales reached TOC contents of up to 36.23% and relatively high TS content between 1.46 and 2.38 wt.%, which could be attributed to good preservation under reducing paralic environmental conditions. These reducing paralic environmental conditions are consistent with the dominant presence of marine *telalginite* and *lamalginite* algae, with low contributions of freshwater algae, i.e., *Botryococcus,* thus, enhanced growth of algae because of the presence of nutrients.
2. The low values of isoprenoid ratios such as Pr/Ph, Pr/*n*-C_17_, and Ph/*n*-C_18_ further suggest high contributions of phytoplankton algae, deposited under reducing environmental conditions.
3. The analyzed bituminous shales contain high levels of aquatic-derived organic matter, with mainly phytoplankton algae of marine origin, are rich in Type II kerogen, with HI values and H/C ratios in the range of 352–544 mg HC/g TOC and 1.14–1.38%, respectively. Thus, these shale sediments could be oil-shale resources.
4. The optical and chemical maturity data confirmed that the analyzed bituminous shales contain immature organic matter, and the hydrogen-rich Type II kerogen oil has not yet cracked to oil at the current thermally immature stage.
5. These findings improve the opportunities and unconventional oil-shale resource exploration approaches in the Bikaner sub-basin of Rajasthan state, NW India. Artificial heating is required to extract commercial quantities of oil from the high bituminous shales in the Gurha mine, NW India.
